# Factors associated with persistent posttraumatic stress disorder among U.S. military service members and veterans

**DOI:** 10.1186/s12888-018-1590-5

**Published:** 2018-02-17

**Authors:** Richard F. Armenta, Toni Rush, Cynthia A. LeardMann, Jeffrey Millegan, Adam Cooper, Charles W. Hoge, for the Millennium Cohort Study team

**Affiliations:** 1 Deployment Health Research Department, Naval Health Research Center, 140 Sylvester Road, San Diego, CA 92106-3521 USA; 2 The Henry M. Jackson Foundation for the Advancement of Military Medicine, Inc, Bethesda, MD USA; 3 Department of Family Medicine and Public Health, La Jolla, University of California, San Diego, School of Medicine, San Diego, CA USA; 4 Directorate of Mental Health, Naval Medical Center San Diego, San Diego, CA USA; 5 Walter Reed Army Institute of Research, Silver Spring, MD USA

**Keywords:** Post-traumatic stress disorder, PTSD, Military, Combat, Veterans

## Abstract

**Background:**

Posttraumatic stress disorder (PTSD) can have long-term and far-reaching impacts on health and social and occupational functioning. This study examined factors associated with persistent PTSD among U.S. service members and veterans.

**Methods:**

Using baseline and follow-up (2001–2013) questionnaire data collected approximately every 3 years from the Millennium Cohort Study, multivariable logistic regression was conducted to determine factors associated with persistent PTSD. Participants included those who screened positive for PTSD using the PTSD Checklist–Civilian Version at baseline (*N* = 2409). Participants were classified as having remitted or persistent PTSD based on screening negative or positive, respectively, at follow-up.

**Results:**

Almost half of participants (*N* = 1132; 47%) met criteria for persistent PTSD at the first follow-up; of those, 804 (71%) also screened positive for PTSD at the second follow-up. Multiple factors were independently associated with persistent PTSD in an adjusted model at the first follow-up, including older age, deployment with high combat exposure, enlisted rank, initial PTSD severity, depression, history of physical assault, disabling injury/illness, and somatic symptoms. Among those with persistent PTSD at the first follow-up, additional factors of less sleep, separation from the military, and lack of social support were associated with persistent PTSD at the second follow-up.

**Conclusions:**

Combat experiences and PTSD severity were the most salient risk factors for persistent PTSD. Comorbid conditions, including injury/illness, somatic symptoms, and sleep problems, also played a significant role and should be addressed during treatment. The high percentage of participants with persistent PTSD supports the need for more comprehensive and accessible treatment, especially after separation from the military.

## Background

Posttraumatic stress disorder (PTSD) is one of the most common health conditions among U.S. service members, with an estimated prevalence between 5% and 20% among the 2.7 million who have deployed to Iraq and Afghanistan since 2001, depending primarily on level of combat exposure [1, 2]. PTSD has been associated with a nearly 200% increase in hospitalizations among active duty service members between 2006 and 2012, and is a leading diagnosis in Department of Veterans Affairs medical settings [3]. However, these statistics underestimate the impact of PTSD since many service members in need of treatment do not seek care [1].

The course of PTSD varies, but symptoms can become persistent and last years or even a lifetime [4–7]. Persistent PTSD has been associated with long-term neurobiological changes and comorbidities that can have profound effects on physical and mental health functioning [8–11]. These changes may affect the person’s psychological, behavioral, and physical health [12–14], decreasing one’s quality of life and manifesting in the form of increased rates of cardiovascular disease [12, 15], hypertension, obesity [12, 16], immune-mediated disorders [17], and mortality. In addition, PTSD often co-occurs with depression, anxiety, and substance abuse [1, 18], all of which have been linked to suicidal behaviors [19]. As a direct effect of PTSD or an indirect result of comorbid health effects, important social interactions involving intimate relationships, such as marriage, are also often negatively affected, leading to decreased support and further worsening of symptoms [20]. Ongoing PTSD has been associated with increased disability, decreased productivity, and decreased fitness, thereby limiting one’s ability to continue to serve in the military or function in other occupational settings [21]. The longer PTSD remains untreated, the greater the probability that this stress-related disorder will lead to a health complication [22]. Therefore, understanding the mechanisms related to the persistence of PTSD symptoms is critical to the life and well-being of service members, veterans, and their families.

Limited research has examined factors associated with persistent PTSD among U.S. veterans of the Vietnam conflict [23–25] and World War II [26], as well as British veterans of the recent Iraq conflict [27]. In these studies, combat severity was associated with higher occurrence of PTSD and persistence of symptoms [23–26]. Other notable factors associated with persistent PTSD included lack of social support [23, 26, 27], preexisting depression [24], and reporting of multiple physical symptoms [13, 27]. Other studies have found that PTSD symptoms may continue to increase over time [4]. Studies that are more comprehensive involving U.S. cohorts and a more comprehensive range of potential risk and protective factors are lacking. Understanding the broad interactions of various risk factors in the development of persistent PTSD among U.S. military personnel is essential to providing specialized multidisciplinary treatment for service members and veterans.

This study is the first, to our knowledge, to prospectively assess persistent PTSD symptoms among U.S. military personnel serving since the start of operations in Iraq and Afghanistan. The primary objective of this study was to estimate rates of persistent PTSD and identify factors associated with persistent symptoms among participants in the Millennium Cohort Study, a large cohort study of service members and veterans from all service branches and components [28, 29].

## Method

Launched in 2001, the Millennium Cohort Study was designed to examine the impact and long-term health effects associated with military service. Between 2001 and 2013, four separate groups of service members, referred to as panels, were enrolled. Participants were randomly selected from U.S. military personnel and asked to complete a comprehensive web-based or paper survey approximately every 3 years. A detailed description of the Millennium Cohort Study methodology has been published elsewhere [28, 29].

The present study included Millennium Cohort participants who enrolled in the first or second panel. Participants must have completed at least two questionnaires, the 2004–2006 and 2007–2008 surveys, approximately 3 years apart (*N* = 63,589). Data from the 2004–2006 survey cycle and 2007–2008 survey cycle are referred to as “baseline” and “first follow-up,” respectively, for the purposes of this paper. To be eligible for this analysis, participants must have screened positive for PTSD at baseline (*N* = 2992). After excluding those with missing data for the key outcome or predictor variables, the study population between baseline and the first follow-up included 2409 individuals (80.5% of the eligible sample). In addition, participants who screened positive for PTSD at first follow-up had complete data for the covariates of interest, and those who had follow-up data at the subsequent 2011–2013 survey cycle (*N* = 822) were assessed for persistent PTSD at the “second follow-up.”

### Outcome

PTSD was assessed using the PTSD Checklist–Civilian Version (PCL-C), a 17-item measure that asks participants to self-report the severity of each item in the past month. PCL-C responses are scored with a 5-point Likert scale ranging from 1 (“not at all”) to 5 (“extremely”), with a possible total score of 17 to 85. Based on the DSM-IV-TR, participants screened positive for PTSD if they reported a moderate or higher level (scoring ≥3) for at least one intrusion, at least three avoidance, and at least two hyperarousal symptoms [30]. This DSM-IV-TR criterion for scoring the PCL-C has been shown to correspond to a total cutoff of 44 in military personnel, a sufficiently high threshold for estimating prevalence in a population study [31, 32]. Participants who met these criteria at follow-up were defined as having persistent PTSD, and those who no longer screened positive were defined as having remitted. A continuous PTSD severity score at baseline was also included in analyses to determine whether those with higher initial PTSD scores were more likely to have persistent PTSD. PTSD severity scores were modeled as a 10 point change based on the standard deviation and what might be considered a clinically relevant change in scores.

### Covariates

Demographic and military characteristics were obtained at baseline from personnel files from the Defense Manpower Data Center and included sex, birth cohort, race/ethnicity, marital status, educational level, service branch, military component, pay grade, military separation, and military occupation. All other covariates, with the exception of deployment, were measured at baseline for the first analysis (i.e., persistent PTSD assessed at the first follow-up) and at the first follow-up for the second analysis (i.e., persistent PTSD assessed at the second follow-up).

Deployment experience in support of the operations in Iraq and Afghanistan prior to the first follow-up was determined using Department of Defense electronic military records. Combat experiences were assessed at the first follow-up using a 12-item modified version of the Walter Reed Combat Experiences Scale (e.g., being attacked or ambushed, seeing dead bodies, knowing someone seriously injured or killed), with an aggregate score of 1–12 representing more variation in type of combat experiences. Deployment and combat experience/severity were combined into one variable with five categories reflecting no deployment, deployment without combat, and deployment with low combat (score, 1–3), medium combat (score, 4–10), and high combat (score, > 10), using methods similar to prior studies [1].

Depression was assessed by self-reporting “ever” being diagnosed with depression by a medical professional or screening positive on the 8-item Primary Care Evaluation of Mental Disorders Patient Health Questionnaire depression scale (PHQ-8) [33]. Based on DSM-IV-TR criteria, participants screened positive for depression if they met the following criteria: responded “more than half the days” or “nearly every day” to at least 5 of the 8 items, in which one of the endorsed items was a depressed mood or anhedonia. Participants were also asked if they had ever been diagnosed with manic disorder or schizophrenia, or had been prescribed a medication to treat anxiety, panic, or depression.

Functional physical health was assessed using the physical component summary score derived from the Medical Outcomes Study Short Form 36-Item Health Survey for Veterans [34]. Continuous scores, in which higher scores represent better health, were collapsed into three categories; consistent with previous methodology, “low” and “high” represented scores below the 15th percentile and above the 85th percentile, respectively [35]. Multiple physical symptoms were assessed using the 15-items from the Patient Health Questionnaire (PHQ-15) [36]. The first 13 items are rated on a 0 (“not bothered at all”) to 2 (“bothered a lot”) scale for the past month; the remaining two items, sleeping trouble and fatigue, are rated on a 0 to 3 scale for the past 2 weeks and were adapted to the 0 to 2 response scale using the validated approach of Kroenke et al. [36]. Scores were summed, and a cutoff score of 15 was used to indicate impairment. A score of ≥15 has been shown to be significantly associated with increased impairment and higher utilization of health care services, and has been used in other military studies [13].

Behavioral characteristics (e.g., body mass index [BMI], sleep, tobacco use, alcohol use) were based on self-reported data assessed at baseline. BMI was categorized as underweight (< 18.5 kg/m^2^), normal weight (18.5–24.9 kg/m^2^), overweight (25.0–29.9 kg/m^2^), and obese (≥ 30 kg/m^2^); underweight and normal weight were combined due to low numbers of underweight individuals. Sleep duration was categorized into three groups: < 4 h, 4–6 h, and > 6 h. Tobacco use was categorized as current smoker, past smoker, and nonsmoker, and based on questions about ever smoking 100 cigarettes, current smoking, and successfully quitting smoking. Alcohol-related problems were assessed using the five PHQ items and based on one or more affirmative responses [37]. Social support was measured by how much participants reported being bothered by “having no one to turn to when they have a problem” in the last 4 weeks, and dichotomized to “not bothered at all/bothered a little” and “bothered a lot.” Each stressful life experience was assessed as a binary variable based on endorsement of divorce, financial problems, physical assault, sexual assault, illness or death of a loved one, and personal disabling illness or injury. This study was approved by the Institutional Review Board at the Naval Health Research Center and all participants provided voluntary written informed consent.

### Statistical analysis

Descriptive analyses and chi-square tests were used to compare demographic, behavioral, and military characteristics by PTSD persistence. Bivariate logistic regression was conducted to determine factors associated with persistent PTSD. Multivariable logistic regression models, for both the first and second follow-ups, were derived using a backwards elimination process that initially included all variables associated with PTSD in the unadjusted models (*p* < 0.20) [38, 39]. Factors were manually removed sequentially until the final model included only those that were significantly associated with PTSD (*p* < 0.05) or confounded the relationship between any covariate and PTSD by >10% [40]. Collinearity was assessed using variance inflation factors and condition indices. All analyses were conducted using SAS software, version 9.3 (SAS Institute Inc., Cary, N.C.).

## Results

Of the 2409 service member participants who met criteria for PTSD at baseline, the mean age was 32.2 years (SD = 10.2), 65% were male, and 71% were non-Hispanic white. 49% of the sample deployed between 2001 and the first follow-up visit; of those, 90% reported experiencing combat.

Overall, 1123 (47%) service members had persistent PTSD at the first follow-up (2007–2008). In bivariate analyses (Table 1), numerous characteristics were significantly associated (all *p* < 0.05) with persistent PTSD, including birth year prior to 1960 (vs. 1980 or later), lower educational level, being married, being in the Army or Marine Corps, having an enlisted pay grade, having a history of deployment with moderate to severe combat, reporting greater PTSD severity at baseline, presence of another mental health condition (i.e., depression, manic disorder, schizophrenia), use of psychotropic medication, higher BMI (i.e., obese), lower physical functioning, screening positive for multiple physical symptoms, short sleep duration (≤ 4 h), being a current smoker, and history of stressful experiences (e.g., sexual assault, divorce, physical assault, disabling illness or injury).

**Table 1:** Frequencies and Unadjusted Odds Ratios of Characteristics Measured in 2004–2006 by Persistent PTSD at the First Follow-Up for Millennium Cohort Participants (*N* = 2409)

Variables	Persistent PTSD					
	No (*N* = 1286)		Yes (*N* = 1123)		OR	95% CI
Demographics	Number	Percent	Number	Percent		
Sex						
Male	823	64.0	742	66.1	1.0	
Female	463	36.2	381	33.9	0.91	0.77–1.08
Birth year*						
Prior to 1960	145	11.3	172	15.3	1.60	1.23–2.10
1960–1969	326	25.4	286	25.5	1.19	0.95–1.48
1970–1979	419	32.6	372	33.1	1.20	0.98–1.47
1980 or later	396	30.8	293	26.1	1.0	
Education*						
Some college or less	1019	79.2	936	83.3	1.0	
Bachelor’s degree or higher	267	20.8	187	16.7	0.76	0.62–0.94
Race/ethnicity						
Non-Hispanic White	928	72.2	781	69.5	1.0	
Non-Hispanic Black	132	10.3	146	13.0	1.31	1.02–1.69
Other	226	17.6	196	17.5	1.03	0.83–1.28
Marital status*						
Never married	532	41.4	403	35.9	1.0	
Married	615	47.8	587	52.3	1.26	1.06–1.50
Other	139	10.8	133	11.8	1.26	0.96–1.66
Military Characteristics						
Service branch*						
Army	753	58.6	722	64.3	1.44	1.14–1.81
Navy/Coast Guard	216	16.9	162	14.4	1.12	0.84–1.51
Marine Corps	95	7.4	91	8.1	1.44	1.01–2.05
Air Force	222	17.3	148	13.2	1.0	
Service component						
Active duty	656	51.0	6594	52.9	1.08	0.92–1.27
Reserve/National Guard	630	49.0	529	47.1	1.0	
Pay grade*						
E1–E4	607	46.2	556	49.5	1.66	1.27–2.17
E5–E9	496	38.6	466	41.5	1.70	1.29–2.24
Officer	183	14.2	101	9.0	1.0	
Military separation						
No	1038	80.7	877	78.1	1.0	
Yes	248	19.3	246	21.9	1.17	0.96–1.43
Military occupation						
Combat	231	18.0	217	19.3	0.96	0.73–1.35
Health care	133	10.3	126	11.2	1.0	
Other	922	71.7	780	69.5	0.89	0.69–1.16
Deployment experience^a,^*						
Nondeployed	675	52.5	555	61.4	1.0	
Deployed without combat	91	7.1	28	5.2	0.34	0.22–0.54
Deployed, low combat	162	12.6	73	6.5	0.55	0.41–0.74
Deployed, moderate combat	320	24.9	369	32.9	1.41	1.17–1.70
Deployed, high combat	38	2.9	98	8.7	3.13	2.12–4.63
Medical Characteristics						
PTSD severity at baseline (mean, SD per 10-point change in score)^b,^*	50.8	9.7	57.6	11.3	1.84	1.70–2.01
Depression^c,^*						
No	538	41.8	277	24.7	1.0	
Yes	748	58.2	846	75.3	2.20	1.84–2.62
Manic*						
No	1238	96.3	1025	91.3	1.0	
Yes	48	3.7	98	8.7	2.47	1.73–3.52
Schizophrenia*						
No	1271	98.8	1090	97.1	1.0	
Yes	15	1.2	33	2.9	2.56	1.39–4.75
Psychotropic medication^d,^*						
No	1024	79.6	767	68.3	1.0	
Yes	262	20.4	356	31.7	1.81	1.51–2.18
BMI categories^e,^*						
Under/normal weight	457	35.5	365	32.5	1.0	
Overweight	582	45.3	486	43.3	1.04	0.87–1.26
Obese	247	19.2	272	24.2	1.38	1.11–1.72
Physical component score*						
Low	148	11.5	229	20.4	1.0	
Mid	907	70.5	760	67.7	0.54	0.43–0.68
High	231	18.0	134	11.9	0.37	0.28–0.50
Multiple somatic symptoms score*						
< 15	1079	83.9	778	69.3	1.0	
≥ 15	207	16.1	345	30.7	2.31	1.90–2.81
Behavioral Characteristics						
Sleep duration*						
< 4 h/day	230	17.9	310	27.6	1.41	1.11–1.78
4–6 h/day	358	27.8	368	23.9	1.15	0.82–1.61
> 6 h/day	698	54.3	545	48.5	1.0	
Smoking*						
Past smoker/nonsmoker	934	72.6	778	69.2	1.0	
Current smoker	352	27.4	3345	30.7	1.17	0.98–1.40
Alcohol-related problems						
No	960	74.6	826	73.5	1.0	
Yes	326	25.4	297	26.5	1.06	0.88–1.27
Stressful Experiences						
Divorced*						
No	778	60.5	600	53.4	1.0	
Yes	508	39.5	523	46.6	1.33	1.14–1.57
Financial problems*						
No	923	71.8	765	68.1	1.0	
Yes	363	28.2	358	31.9	1.19	1.00–1.42
Sexual assault*						
None	893	69.4	722	64.3	1.0	
Harassment	158	12.3	149	13.3	1.17	0.91–1.49
Assault	235	18.3	252	22.4	1.33	1.08–1.62
Physical assault*						
No	1032	80.3	791	70.4	1.0	
Yes	254	19.7	332	29.6	1.70	1.41–2.06
Ill/Death of a loved one*						
No	326	25.3	229	20.4	1.0	
Yes	960	74.7	894	79.6	1.33	1.09–1.61
Disabling illness/injury*						
No	989	76.9	725	64.6	1.0	
Yes	297	23.1	398	35.4	1.82	1.53–2.19

*BMI* =body mass index, *CI* =confidence interval, *OR* =odds ratio, *PHQ* =Patient Health Questionnaire, *PTSD* =posttraumatic stress disorder

^a^Deployments include those which occurred prior to the 2007 survey date and were in support of operations in Iraq and Afghanistan

^b^PTSD severity at baseline was included as a continuous variable to adjust for varying PTSD scores. Results were modeled using a 10 point change in the severity score based on the standard deviation and what would be considered a clinically relevant change the PTSD score

^c^Depression includes diagnosis by a medical professional or screening positive on the PHQ-9

^d^Endorsed “yes” to survey question, “Are you currently taking any medicine for anxiety, depression, or stress?”

^e^BMI categories: under/normal weight (≤24.9 kg/m2), overweight (25.0–29.9 kg/m2), and obese (≥30 kg/m2)

^*^Variable significant at p < 0.20 using chi-square testing

In the final multivariable model (Table 2), factors that remained significantly associated with persistent PTSD at the first follow-up period (2007–2008) included older age, enlisted pay grade (with junior enlisted pay grade having highest odds), deployment with moderate or higher combat experiences, higher baseline PCL-C score, multiple physical symptoms (PHQ-15 score ≥ 15), depression, prior experience of physical assault, or disabling injury/illness. High combat exposure (OR = 3.91) and severity of PTSD symptoms at baseline (OR = 1.59 for each 10-point increment on the PCL) were the strongest risk factors for persistent PTSD.

**Table 2:** Multivariable Regression Analysis of Predictors of Persistent PTSD at the First (2007–2008) and Second Follow-Ups (2011–2013)

	First Follow-Up (*N* = 2409) (47% With Persistent PTSD)		Second Follow-Up (*N* = 822) (71.2% With Persistent PTSD)	
Characteristic*	OR	95% CI	OR	95% CI
Birth year				
Prior to 1960	2.34	1.68–3.54	2.68	1.53–4.68
1960–1969	1.71	1.25–2.36	2.11	1.32–3.36
1970–1979	1.42	1.11–1.83	1.40	0.91–2.14
1980 or later	1.00		1.00	
Pay grade				
Junior enlisted (E1–E4)	2.10	1.50–2.93		
Senior enlisted (E5–E9)	1.68	1.25–2.27		
Officer	1.00			
Deployment experience^a^				
Nondeployed	1.00		1.00	
Deployed without combat	0.56	0.36–0.90	0.91	0.33–2.51
Deployed, low combat	0.73	0.53–1.00	1.77	0.90–3.50
Deployed, moderate combat	1.72	1.39–2.12	1.44	0.98–2.12
Deployed, high combat	3.91	2.56–5.96	2.63	1.27–5.42
PTSD severity at baseline^b^				
Per 10 point change in score	1.59	1.45–1.74	1.23	1.06–1.43
Multiple somatic symptoms score				
< 15	1.00			
≥ 15	1.47	1.18–1.83		
Depression^c^				
No	1.00		1.00	
Yes	1.50	1.23–1.83	2.33	1.63–3.33
Physical assault				
No	1.00			
Yes	1.25	1.01–1.54		
Sleep duration				
< 4 h per day			1.75	1.15–2.69
4–6 h per day			1.08	0.74–1.58
> 6 h per day			1.00	
Separation from military				
No			1.00	
Yes			1.57	1.12–2.21
Social support				
Not bothered/bothered a little			1.00	
Bothered a lot			1.51	1.09–2.10
Disabling injury/illness				
No	1.00			
Yes	1.35	1.11–1.66		

*CI* =confidence interval, *OR* =odds ratio, *PHQ* =Patient Health Questionnaire, *PTSD* =posttraumatic stress disorder

^a^Deployments include those which occurred any time prior to the 2007 survey date and were in support of operations in Iraq and Afghanistan

^b^PTSD severity at baseline was included as a continuous variable to adjust for varying PTSD scores. Results were modeled using a 10 point change in the severity score based on the standard deviation and what would be considered a clinically relevant change the PTSD score

^c^Depression includes diagnosis by a medical professional or screening positive on the PHQ-9

*All variables with OR and 95% CI are significant at the alpha < 0.05 level

Of the 1792 participants who screened positive for PTSD at baseline and had available data from the three survey cycles, 592 (33.0%) screened positive for PTSD on both follow-up questionnaires. Among those who screened positive for PTSD at the first follow-up and had available data at the second follow-up (*N* = 822), 585 (71.2%) continued to screen positive at the second follow-up visit. In addition to many of the aforementioned factors associated with persistent PTSD, separation from the military, sleeping less than 4 h per night, and lack of social support were associated with persistence of PTSD at the second follow-up (Table 2).

## Discussion

PTSD is one of the most common disabling disorders among U.S. service members and veterans, and it often becomes a chronic condition. In this study, we found that 47% of those who screened positive for PTSD at baseline were still positive for PTSD at follow-up approximately 3 years later; of those, over 70% remained positive for PTSD at the second follow-up approximately 6 years later.

Results from this study indicate that the intensity of combat exposure during the recent operations in Iraq and Afghanistan is a strong risk factors for persistent PTSD. This finding is consistent with other research that suggests combat exposure, compared with other types of trauma, confers a uniquely high risk of persistent PTSD, with strong linear associations related to combat intensity [2, 27, 41, 42]. Conversely, individuals who deployed without experiencing combat were more likely to screen negative for PTSD at follow-up compared with those who did not deploy. This protective association among non-combat deployers is likely due to the well-known healthy warrior effect, in which healthier service members are more likely to deploy than service members experiencing medical problems [43].

In addition to combat intensity, a number of other important risk factors for PTSD chronicity were identified, in particular indicators of psychological or physical health comorbidities. Not surprisingly, PTSD severity at baseline was strongly associated with persistence of symptoms. However, even after adjusting for PTSD severity and other variables, comorbid health conditions, which are common among those with PTSD, still emerged as important and independent risk factors of persistent PTSD. These findings indicate that those with comorbid conditions, such as depression or multiple physical symptoms, may be different in some ways than those with the single condition of PTSD. These comorbid conditions a may lead to increased dysregulation of neuroendocrine and autonomic nervous system or interoceptive functions. These comorbid conditions could also be linked to pain perception changes or physical symptoms acting as reminders of the traumatic event, either directly from injury that occurred as part of the trauma or indirectly through physiological mechanisms associated with bodily sensations [44]. For these and other reasons, it may be more difficult to effectively treat and care for individuals with PTSD who have other additional conditions.

One notable finding was that participants who reported sleeping less than 4 h per night at the first follow-up survey were more likely to screen positive for PTSD at the second follow-up, suggesting that sleep is an important predictor of long-term persistent PTSD. Sleep problems are common among service members who have deployed, independent of PTSD, and research has shown that those who develop disruptions in normal sleep patterns after a traumatic event are more likely to develop PTSD [45]. Additionally, sleep problems may result from the PTSD symptoms themselves, such as hypervigilance and recurrent nightmares. Sleep problems are also associated with comorbid conditions. Given these multiple interrelated health associations, treatment of persistent PTSD symptoms may include comprehensive approaches that address comorbid physical health problems, chronic pain, and sleep problems [44, 46].

An important aspect of PTSD treatment is having adequate social support. We found that being bothered a lot by not having someone to turn to was associated with persistent PTSD at the second follow-up. Research shows that social relationships can help with treatment compliance, coping with intrusive symptoms, and sharing feelings and thoughts that are experienced with PTSD. Social support both during and following deployment has been shown to be associated with lower PTSD severity [47]. Additionally, separation or retiring from the military was associated with persistent PTSD between the first and second follow-up visits, which may also relate in part to changes in social relationships after transitioning from the military. It is also likely that persistent PTSD itself led to medical discharge from the military for some study participants [48].

Participants born before 1980 were more likely to have persistent PTSD. Military studies have generally observed that the risk of PTSD is highest in younger age groups; however, when examining persistence or chronicity, studies have more often shown an association with older age [27, 49]. Studies among aging war veterans and Holocaust survivors have reported that PTSD severity increases in older trauma survivors [49].

We did not find an association between reported history of sexual trauma and persistent PTSD in adjusted models, though there was an association in unadjusted models. Sexual assault has been shown to be one of the most significant types of traumatic events in predicting PTSD and other mental health outcomes [50, 51]. In our analysis, the null association with sexual assault may be attributable to the small number of sexual assault cases or high correlation with other statistically significant variables such as physical assault. It is also possible that the measure of sexual assault was less than optimal and thus could have missed some cases of sexual assault or lead to misclassification. Further research to examine the role of sexual trauma in PTSD persistence is needed.

### Study limitations and strengths

The results of our study must be interpreted with some limitations in mind. The study population consisted of a sample of responders to the Millennium Cohort questionnaire and may not be representative of the overall military population. However, investigations of potential biases have found the cohort to be highly representative, without evidence that participation is influenced by poor health or other key factors that would relate to health outcomes analyses [52, 53]. Self-reported survey data may be subject to recall and reporting biases; however, there was high consistency in associations between time periods. It is still possible that participants could inaccurately recall information across time periods. While the PCL-C is a screening tool for and not diagnostic of PTSD, it has strong demonstrated validity, and the associations with PCL severity found in this study were consistent with other research [31]. The follow-up period (3 years between surveys) was relatively long, and we were not able to study PTSD treatment received in military, veteran, or civilian settings by cohort participants. Thus, it was not possible to look at the trajectory of fluctuations in symptomatology over shorter periods of time, the effectiveness of treatment that may have been received, or other factors related to service utilization, such as receiving medical disability for PTSD or related health conditions. We did not assess lifetime or childhood trauma history, which may increase the risk of persistent PTSD; this should be included in future studies. Our assessment of social support was limited to one item, thus further studies should include a more comprehensive measure of social support to better understand how it is associated with persistent PTSD. Finally, 19.5% of participants were excluded due to missing data. In examining those with missing data compared to those with no missing data for all covariates we found that only three (sex, birth cohort, and race/ethnicity) were statistically different, albeit the differences were less than 5%. Further, In a bivariate sensitivity analysis using all data available for race, sex, and birth cohort there were no significant differences in associations with persistent PTSD when compared with bivariate analysis using only observations with complete data (data not shown). These additional analyses indicated that the missingness likely did not impact the results.

Despite these limitations, this study has many notable strengths. To our knowledge, this is the first study to longitudinally examine persistent PTSD among a large cohort of U.S. service members and veterans. The sampling method was population based and represents all service branches. The study methodology using the PCL-C allowed for the detection of individuals suffering from PTSD who may not seek care [1]. Finally, due to the prospective and comprehensive nature of the survey, we were able to examine multiple risk factors.

## Conclusions

Understanding correlates of PTSD chronicity is critical to the long-term health and well-being of service members and veterans. Almost half of participants who screened positive for PTSD at baseline had persistent PTSD at the first-follow-up; among those, over two thirds had persistent PTSD at the second follow-up. Given the large number of factors associated with PTSD persistence, our study highlights the importance of holistic collaborative care models that address complex interrelated comorbid health conditions, including depression, generalized physical symptoms, and sleep problems [32].
